# Evidence-based approach to glaucoma management

**DOI:** 10.4103/0301-4738.73680

**Published:** 2011-01

**Authors:** Chandrasekhar Garudadri, Sirisha Senthil, Harsha Laxmana Rao

**Affiliations:** VST Center for Glaucoma, LV Prasad Eye Institute, Kallam Anji Reddy Campus, LV Prasad Marg, Road No. 2, Banjara Hills, Hyderabad – 500 034, Andhra Pradesh, India

**Keywords:** Critical appraisal, evidence-based medicine, glaucoma

## Abstract

Evidence-based medicine is an evolving new paradigm. With the advent of numerous new diagnostic techniques and therapeutic interventions, one needs to critically evaluate and validate them by appropriate methods before adopting them into day-to-day patient care. The concepts involved in the evaluation of diagnostic tests and therapy are discussed. For delivering the highest level of clinical care, evidence alone is not sufficient. Integrating individual clinical experience and patients’ perspectives with the best available external evidence is essential.

Glaucoma is the second leading cause of blindness worldwide.[1] As clinicians, we not only owe the best possible care to the patients who seek medical help, but also a responsibility toward those undetected and improperly treated. The current status of glaucoma care in the world can be summarized as follows: more than 50% of glaucoma in the community is undiagnosed (in the developing countries this would be higher than 90%),[2,3] more than 50% of those undiagnosed would have seen an eye care provider in the recent past, more than 50% taking medications do not need them (overtreated),[4] and finally noncompliance with the advised medication varies from 5% to 80%.[5] Missed diagnoses in those previously examined by an eye care professional are attributable to the lack of comprehensive evaluation and appropriate clinical skills.[3,6] The recent advances in the imaging of the optic nerve head seek to achieve an early and “objective” diagnosis of glaucoma. One of the reasons for overdiagnosis is using the results of these technologies in isolation and not in the context of a complete clinical picture.[7] Overtreatment (both medical and surgical) could result from the overestimation of the benefits and underestimation of the risks of therapy. There are many advances in medical therapy, lasers and surgery for glaucoma management. One needs to ascertain if these advances really make a difference to patient-centric outcomes and the quality of life of the patients. For example, nonpenetrating glaucoma surgeries (NPGS) are proposed as an alternative to trabeculectomy as they are reportedly associated with fewer complications.[8] But the cost of NPGS is higher, the intraocular pressure (IOP) achieved is not as low as in trabeculectomy, and in case they fail, the success of a future filtering procedure might be compromised. Conflicts of interest of investigators as well as the huge financial stakes involved for the industry promoting these advances and sponsoring the research validating these could result in biases in the published results and the ensuing recommendations.[9–11]

An accurate perspective on the utility of the advances in the diagnosis and management of glaucoma necessitates that the published literature is critically appraised before accepting the recommendations and practicing them. The skills involved in performing an efficient literature search and applying formal rules of evaluation to the literature are new skills that a clinician requires.[12,13]

The aim of this article is neither to elaborate on the principles of critical appraisal, nor present a summary of the existing literature on the diagnosis and management of glaucoma. We shall try and summarize the need for the skills for the practice of Evidence-Based Medicine (EBM) in glaucoma diagnosis and management with a few examples. Understandably, this will reflect our own perspective and biases in glaucoma management. As Sweeney said, “it is almost impossible for doctors to be clinically dispassionate or completely neutral about a topic.”[14]

Sackett *et al*. defined EBM as “integrating individual clinical expertise with the best available external clinical evidence.”[15] The evidence probably has two levels of hierarchies. The first level comprises clinical decision support systems at the highest level, followed by synopses, syntheses, and finally individual studies. At the lower level, there are individual studies, with a hierarchy ranging from meta-analysis of randomized controlled trials (RCTs), a single RCT, observational uncontrolled studies, and case reports.[12] The Cochrane database of syntheses and the few meta-analyses do provide the best possible evidence but unfortunately do not address all the clinical issues in ophthalmology. Thus our practice of EBM is significantly limited to single RCTs and observational studies. We need to realize that the processes of peer review with its best intentions can sometimes fail,[16] thus reinforcing the need for skills in formal rules of appraisal of evidence.

## Diagnosis

**Figure 1:**
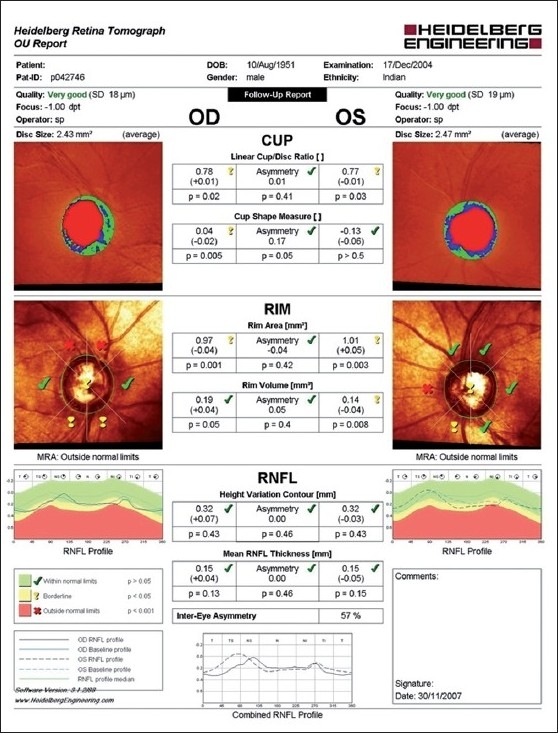
Heidelberg retina tomograph showing an abnormal result in both the eyes

**Figure 2:**
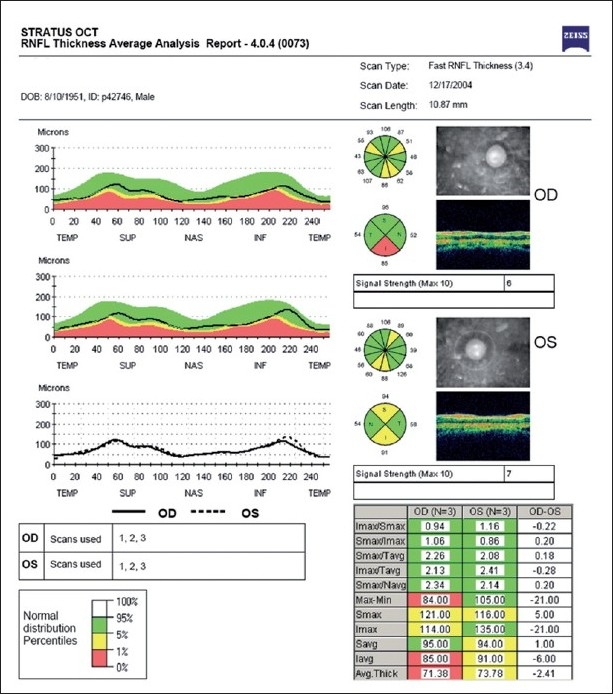
Optical coherence tomograph (OCT) showing an abnormal result in both the eyes

**Figure 3:**
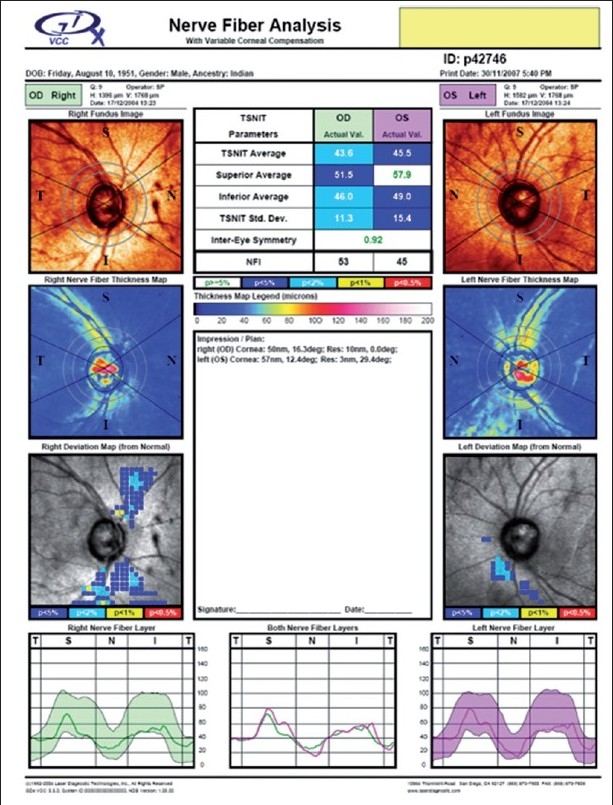
GDx showing an abnormal result in both the eyes

**Figure 4:**
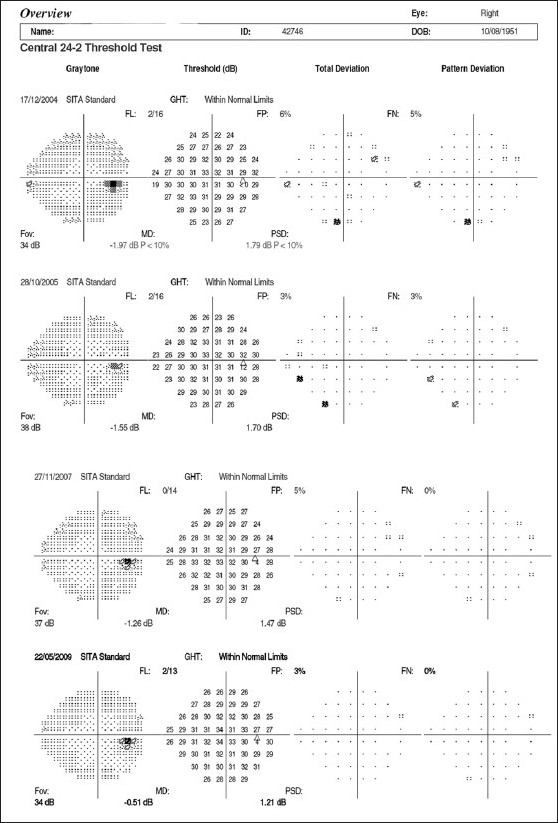
Normal Humphrey visual fields of the right eye

**Figure 5:**
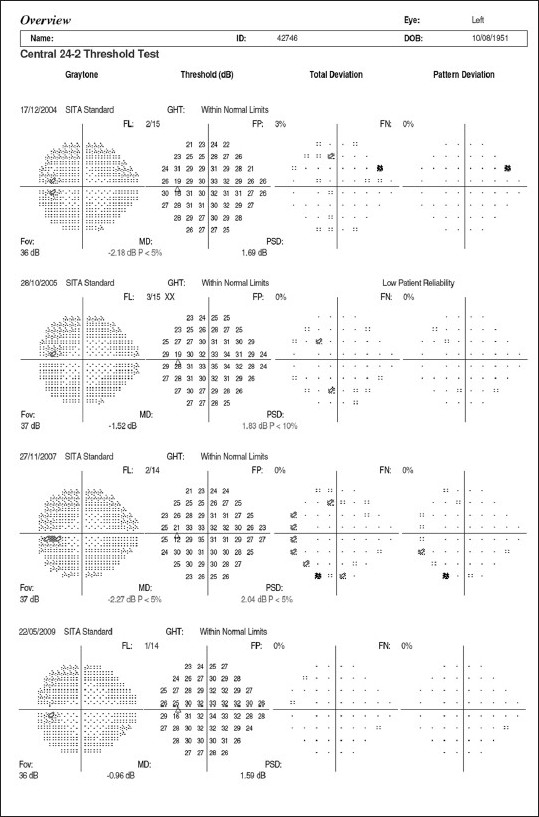
Normal Humphrey visual fields of the left eye

Imaging of the optic nerve head and retinal nerve fiber layer are widely reported to be useful in glaucoma diagnosis.[17] One could (naively) argue that the imaging tests are objective and can be performed easily in uncooperative patients (since they are quick and need little attention) as opposed to standard automated perimetry (SAP), which is the current standard but time consuming and difficult due to the subjective nature of the test. Further, as it is reported that 20–40% retinal ganglion cells could be lost before a visual field defect could be picked up by SAP, these imaging technologies can theoretically aid in the early diagnosis of glaucoma. This rationale need not necessarily convert to a clinical reality. Figs. 1–5 show a glaucoma suspect (because of a positive family history and suspicious optic disc) followed up for a decade. All the three glaucoma imaging technologies showed an “abnormality,” but the SAP result was always normal. Why does this happen? The reported sensitivity and specificity of these technologies and their agreement with each other are far from ideal.[18] The sensitivity (ability to diagnose the disease correctly – PID: positive in disease) of the best parameter for each of the imaging technologies at a fixed specificity (NIH: negative in health or ability to declare a normal subject as not having the disease) of 95% (labeling only five as abnormal out of a hundred normal subjects) varies from 36% to 72%. This means that the imaging technologies fail to diagnose glaucoma in as many as 28–64% of the eyes with established abnormality on SAP. While we perform these investigations with the idea of either establishing or ruling out a disease, we fail to realize that there is a cost-benefit ratio even in diagnostic testing. These tests are expensive, there are risks of overdiagnosis with the attendant effect of labeling, and there are problems with interpretation (the normative database of the machine used for diagnostic classification is often limited to a small number of subjects and few ethnicities and hence may not be appropriate to apply for testing patients of all ethnicities).

Garway-Heath appropriately stated that “devices cannot diagnose our patients’ conditions, but the findings they provide frequently alter the probability that a subject has a particular condition.”[6] Thus devices cannot be a substitute for poor clinical skills but at best a complement to good clinical evaluation. If after a good and comprehensive clinical evaluation, the probability (pretest probability) of the disease is around 20%, it is futile to perform any diagnostic test, as even a positive result cannot confirm the presence of the disease. On the other hand, if the probability of the disease is about 80% after a good clinical examination (the need for testing is not to diagnose but probably to establish a baseline for follow-up), even a negative test result cannot rule out the disease. It is in the intermediate range, when the probability of disease is around 50% that the diagnostic test result would strongly help in the diagnostic decision. Before ordering a diagnostic test, it is useful to ask ourselves as to how our course of action would change based on the test result. The probability of the disease before and after the diagnostic test can be mathematically calculated using the likelihood ratio (LR) of the test result in consideration. If we want to use the results of the diagnostic tests appropriately, estimating the LR is the best way around. Thus it is important for us to know the LRs associated with different test results before investing in a new diagnostic test.

The principle behind the use of the LRs is the calculation of the increase in the probability for a given diagnosis given the prior probability of the disease (based on a complete clinical evaluation or the prevalence of the disease) and the result of the diagnostic test with its LRs. This principle is applicable not only for the diagnosis of a disease but also for any event. This principle in mathematical terms is called the Bayesian theorem. Keeping the mathematics aside, the concept is very intuitive and we keep using this in our daily lives extensively, albeit without the mathematical calculations or the complex terminologies.

**Figure 6:**
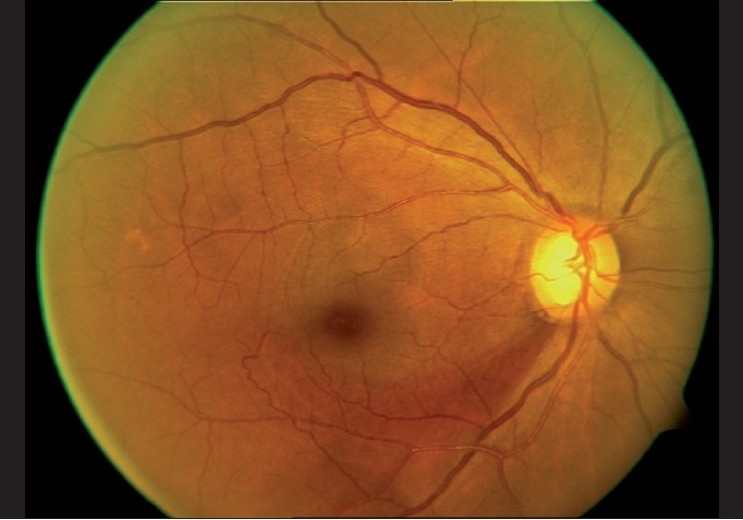
Fundus photograph showing the inferior NFL defect

**Figure 7:**
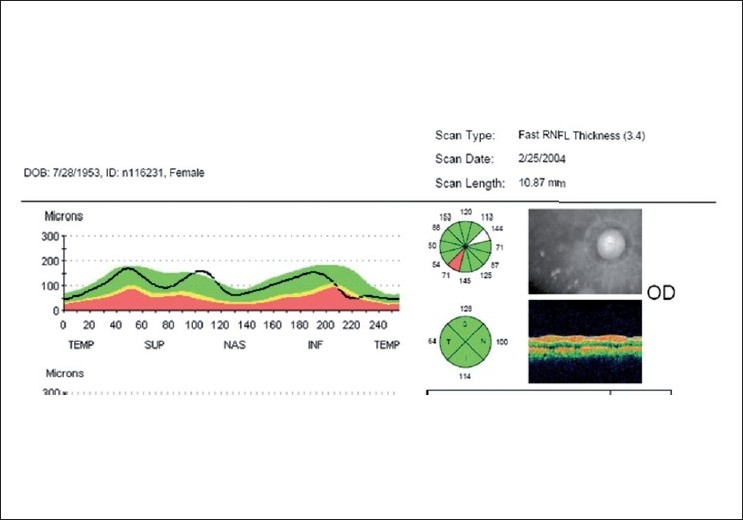
Ocular coherence tomography (OCT) showing inferior NFL loss

**Figure 8:**
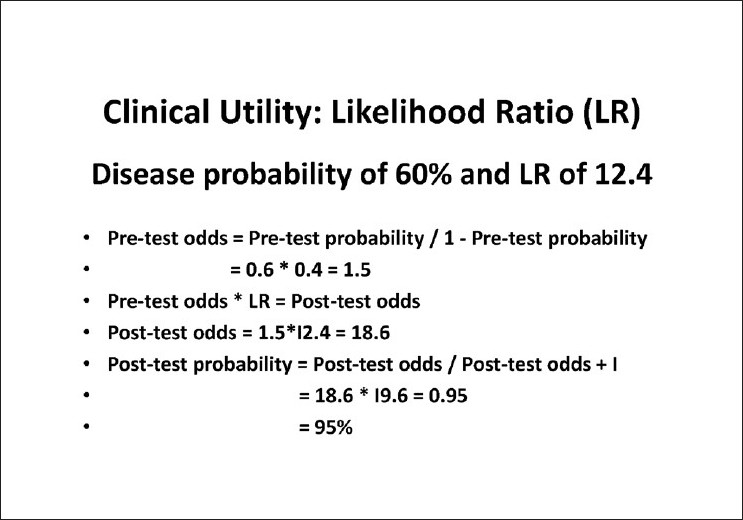
Calculation of the post-test probability using the likelihood ratio of inferior average thickness of ocular coherence tomography (OCT)

For example, on a cloudy day during the rainy season, if it rains we say “it is bound to happen” and if it did not rain, one might say “that was very unlikely” or some such thing. If a 25-year-old, healthy, prospective army recruit ends up with an ST segment change on electrocardiogram (ECG), one would suspect the test or the machine, as the likelihood of this young, healthy man having ischemic heart disease (IHD) is very low. The same result in a 60-year-old gentleman with diabetes and hypertension with precordial discomfort on exertion would be a diagnostic sign of IHD. The prior probability of IHD is very high in the latter and very low in the former. This explains why the same test result is interpreted differently in different situations. The clinical use of the LR is exemplified in Figs. 6–8. The suspicious disc with an inferior nerve fiber layer (NFL) defect on the disc photograph [Figs. 6] and a normal visual field leads us to a prior probability of having glaucoma to be 60%. Integrating the results from ocular coherence tomography [Fig. 7] that shows an abnormality of the inferior nerve fiber layer with the earlier information, the post-test probability of this patient having glaucoma raises to 95%. Fig. 8 shows the actual calculations involved in the above example.

## Therapy

RCTs are the best form of evaluating a therapy. To ensure that the methodological rigor required is followed, there are guidelines for reporting of RCTs called the CONSORT guidelines. How the Ophthalmic literature adheres to these guidelines was evaluated by Lai *et al*. They studied the reporting of 11 key methodological items in RCTs published in American Journal of Ophthalmology, Archives of Ophthalmology, British Journal of Ophthalmology, and Ophthalmology in the year 2005. Among the 67 eligible RCTs assessed, the mean number of items reported was 6.3 per RCT. Details on sequence generation, randomization restriction, allocation concealment, allocation implementation, patient-flow diagrams, and sample size calculation were reported in < 50% of the RCTs assessed. The authors concluded that similar to other specialties, room for improvement exists in the reporting of key methodological items of RCTs in clinical ophthalmic journals. A stricter adoption of the CONSORT statements might enhance the reporting quality of RCTs in ophthalmic journals.[19]

**Figure 9:**
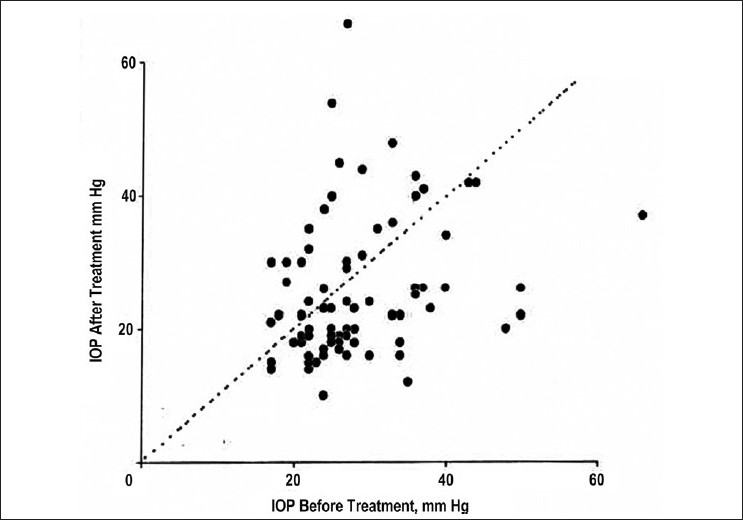
Scatter plot of intraocular pressure (IOP) before and after TSCPC (trans-scleral cyclophotocoagulation). The points above the neutral line indicate that 32.9% (26/79) eyes had higher IOP after therapy compared to before therapy[20]

The possible bias in reporting the study results is exemplified in a study by Egbert *et al*. on the role of primary trans-scleral cyclophotocoagulation in the management of primary open angle glaucoma (POAG).[20] The authors conclude that “the treatment used in the study is free from serious complications, though a new complication of atonic pupil is reported. It is a rapid and easy to learn primary surgical procedure for POAG.”[20] A careful evaluation of the reported results reveals that the reported success rate (20% IOP reduction along with medications) was 48%. If the primary aim of the study is to look for the alternate means of reducing the IOP as the population cannot afford medical therapy, should the definition of outcome measures have been different from the control of IOP along with medications? A careful evaluation of the scatter plot [Fig. 9] from their study shows that the IOP increased from the baseline in 32.9% (26/79) of the eyes. In eyes with vision better than 20/60 preoperatively, vision decreased in 1 out of 19 eyes (5%). Atonic pupil in 29% reported in this series is a new complication of this procedure. Whether it is appropriate to summarize these results as “free from serious complications” needs consideration and not probably just extrapolating the conclusion in the abstract.

In understanding and putting into perspective the results of a therapeutic trial, we need to understand the following terms: relative risk reduction (RRR), absolute risk reduction (ARR), and the number needed to treat (NNT). In the ocular hypertension treatment study (OHTS), the risk of conversion of ocular hypertension (OHT) to glaucoma after a 4-year follow-up was 9.5% in the untreated group and 4.4% in the treated group.[21] The benefit of therapy (ARR) was 5.1%. This risk can be expressed in two other ways. If we consider this reduction as a percentage of the original risk (5.1/9.5), this is approximately 0.5 or 50%. This is the RRR, and the advantage of treating OHT patients according to the RRR of 50% looks huge. If for the sake of discussion, we say that the risk of conversion has reduced from 1% (over 10 years this is approximately the conversion rate for OHT to glaucoma) to 0.5%, the RRR is still 50%. The ARR is 5% in the first example and 0.5% in the second example. The same data can be expressed in terms of NNT, which is the inverse of ARR. In the first example, inverse of 5% will translate to 20, and in the latter, inverse of 0.5% to 200. This means that if we treat 20 patients of OHT for 4 years (achieving a 20% reduction IOP as in the OHTS study), we would prevent one of them from developing early glaucoma as defined in the OHTS study (either disc or visual field change). Just as we say that the cup-to-disc ratio has no meaning in the absence of information on the disc size, RRR has no meaning in isolation without considering the ARR. The same 50% of RRR is obtained if the ARR comes down from 80% to 40% and the NNT in this case would be inverse of 40% or 2.5, meaning that treatment of every two to three patients would benefit one patient, which obviously would strongly favor that treatment.

In the practice of EBM, we should not lose sight of the fact that all these efforts in creating evidence and practicing EBM are for the benefit of the patient. While the best evidence comes from an RCT or a meta-analysis of RCTs, we need to remind ourselves that an RCT is about the intervention and not the patient. If the word *homogeneity* describes the goal of randomization in a clinical trial, then the word *heterogeneity* describes the patient population we see in our practices.[22] We need to exercise caution in extrapolating the results from an RCT to a given patient, whose profile might have excluded him or her from the study. Additionally, while we look at the statistical and clinical significance of the study results, the focus needs to include the patient, described as the third dimension or “personal significance.”[14] A successful communication of the evidence and the advise based on the same is determined not just by the validity of the evidence, but by how this evidence is perceived and communicated by the physician. Equally important is how it is perceived and accepted by the patient. The past experiences and biases of both the patient and the physician strongly influence how the patient–doctor communication gets translated into action and thus the health promotion of the patient. “The core clinical skills of listening, questioning, delineating, marshalling, explaining, and interpreting may provide a way of mediating between the two very different worlds of patients and health professionals. How these tasks are performed is likely to influence the outcome of the illness from the patient’s point of view as much as the scientific and technical aspects of diagnosis or treatment”.[23]

It is important that evidence is not the sole criterion; one needs to compare the risks and benefits, consider the inconvenience and costs as well as patients’ values, and finally the hierarchy of evidence. The importance of EBM should not result in diluting the time-honored craft of caring for the sick. The tools of evidence-based practice are essential but they alone cannot ensure the ability to deliver the highest quality patient care. The highest level of care also requires compassion and an understanding of the patient’s personality, socioeconomic limitations, and cultural values and not just the clinical and surgical skills or the tools of evidence-based practice. Glaucoma is a chronic asymptomatic condition, wherein our understanding of the role of the only treatable parameter (i.e., the IOP) in preventing visual disability is limited.[24] Further, the natural course of the disease is highly variable[25] and the psychological impact of the disease is enormous. Thus, the importance of both the best evidence as well as “craft of caring for the sick” cannot be overestimated.

In conclusion, EBM is a great path to enhance the care of our patients. If we understand the nuances of EBM, we will decide what is best for the individual patient in our care rather than extrapolate the recommendations for the average patient from the most recent RCT as taught by the expert or packaged by the industry.
